# Influence of Al Addition on the Microstructure and Mechanical Properties of Mg-Zn-Sn-Mn-Ca Alloys

**DOI:** 10.3390/ma16103664

**Published:** 2023-05-11

**Authors:** Shujuan Yan, Caihong Hou, Angui Zhang, Fugang Qi

**Affiliations:** 1 National Energy Group Ningxia Coal Industry Co., Ltd., Yinchuan 750001, China; 0411ysj@163.com (S.Y.); 15056070@chnenergy.com.cn (A.Z.); 2 School of Materials Science and Engineering, Xiangtan University, Xiangtan 411105, China; 3 Hunan Bangzer Technology Co., Ltd., Xiangtan 411100, China

**Keywords:** Mg-Zn-Sn-Mn-Ca alloy, Al, microstructure, mechanical properties

## Abstract

The effects of Al addition on the microstructure and mechanical properties of Mg-Zn-Sn-Mn-Ca alloys are studied in this paper. It was found that the Mg-6Sn-4Zn-1Mn-0.2Ca-xAl (ZTM641-0.2Ca-xAl, x = 0, 0.5, 1, 2 wt.%; hereafter, all compositions are in weight percent unless stated otherwise) alloys have α-Mg, Mg_2_Sn, Mg_7_Zn_3_, MgZn, α-Mn, CaMgSn, AlMn, Mg_32_(Al,Zn)_49_ phases. The grain is also refined when the Al element is added, and the angular-block AlMn phases are formed in the alloys. For the ZTM641-0.2Ca-xAl alloy, the higher Al content is beneficial to elongation, and the double-aged ZTM641-0.2Ca-2Al alloy has the highest elongation, which is 13.2%. The higher Al content enhances the high-temperature strength for the as-extruded ZTM641-0.2Ca alloy; overall, the as-extruded ZTM641-0.2Ca-2Al alloy has the best performance; that is, the tensile strength and yield strength of the ZTM641-0.2Ca-2Al alloy are 159 MPa and 132 MPa at 150 °C, and 103 MPa and 90 MPa at 200 °C, respectively.

## 1. Introduction

In this century, the rapid development of automobile, aerospace and other fields has brought a series of problems such as serious environmental pollution and excessive energy consumption. For reducing pollution, reducing energy consumption and improving the living environment, environmentally friendly alloy materials have gradually attracted attention [1]. As a light alloy, the Mg alloy has certain reproducibility and excellent properties such as good casting performance and formability, good shock absorption, good cutting machinability, and good electromagnetic shielding [2,3]. For the past few years, Mg alloys have gradually become the focus of the development and application of new materials [4,5,6]. However, the Mg alloy also has many deficiencies. Firstly, the Mg alloy is easily oxidized at room temperature, and the oxide layer is loose and porous, which greatly limits its application in industrial production. Secondly, the Mg alloy is more active and easy to burn, which makes it necessary to pay attention to the safe production in practical applications. Finally, although the specific strength of the Mg alloy is high, its absolute strength is low, and the high temperature strength is especially poor, which also limits its application. In recent years, many scholars have improved the properties by alloying, that is, by adding a trace or small amount of other elements to the Mg alloy during the cast process.

Due to the addition of different alloying elements, many kinds of alloy systems with different compositions and properties are formed. Among them, the Mg-Zn alloy is a good aging strengthening alloy due to the precipitation of the MgZn_2_ phase [7,8]. The Mg-Sn alloys have a smaller composition span during the smelting process, so that the alloys have fewer defects and better performance [9]. However, it is often not enough to rely on the strengthening effect of a single strengthening phase. In addition, because the strengthening phase is single, the aging strengthening effect is not obvious in the subsequent heat treatment. Therefore, the Mg-Zn and Mg-Sn binary alloy are rarely applied to industrial production. Generally speaking, the alloy’s properties are increased by adding other elements to the binary alloy. The Mg-Zn-Sn alloys are formed by combining the Mg-Zn alloy and the Mg-Sn alloy, and it has the advantages of both alloys. The development of Mg-Zn-Sn alloys have two major advantages: on the one hand is the phenomenon whereby the Mg alloy has poor strength and creep properties at high temperatures because the low melting point phase can be changed. On the other hand, these Mg-Zn-Sn alloys have a wider range of applications, because the price of Zn and Sn elements is lower, and the content on the earth is more, which is convenient for research and development. Therefore, the Mg-Zn-Sn alloys are promising high property deformed magnesium alloys. Based on the existing cognition of the strengthening mechanism of Mg-Zn-Sn alloys, these mechanical properties of Mg-Zn-Sn deformed alloys are further enhanced via adding some other elements or changing some heating treatments. The Mn element can remove harmful elements (such as Fe) in alloy melting, improve the corrosion resistance and casting performance of the alloy, and the Mn element also has a certain ability to refine grain [10].

The Ca element is a common alloying element. The Ca element will nucleate and precipitate on grain boundaries, which inhibit the growth of grains and play the role of grain refinement. At the same time, the Mg_2_Ca phase with good thermal stability is formed when an appropriate amount of Ca is added to the Mg alloy, which can hinder the grain boundaries slip at higher temperature and improve the creep resistance of the alloy [11,12]. It was found that the Mg-Ca alloy has excellent corrosion resistance, high temperature creep performance, and high temperature mechanical properties [13]. Baghani et al. [14] found that when Ca > 2wt.% is added into the alloy, the CaMgSn and Mg_2_Ca phases are generated, which correspondingly reduces the content of the Mg_2_Sn phase. Through this transformation, the properties of the Mg-4Sn alloy are obviously improved. At the same time, many studies have shown that the Ca element should not be too high in the Mg alloy. If the Ca element is too high, the hot cracking tendency will be increased, and the performance will be worse. Therefore, 0.2% Ca element was selected to be added to the alloy in this paper. Al is also a common alloying element. It was found that the appropriate amount of Al will refine the grain and enhance the alloys’ casting properties, and the Mg-Al alloy has good solution strengthening and ageing strengthening effects [15]. Jayaraj et al. [16] found that the Al element will effectively enhance the aging strengthening effect for the Mg-Ca alloy, and then effectively enhance the properties of the Mg-Ca alloy. Wang et al. [17] studied the microstructure and mechanical properties of an as-extruded and aged Mg-Zn-Al-Sn alloy, and found that the comprehensive properties of the Mg-4Zn-2Al-2Sn alloy reached the optimal level after aged treatment at 150 °C for 40 h. Wei et al. [18] researched the effect of different contents of Ca on the microstructure and properties of the Mg-4.5Zn-4.5Sn-2Al alloy, and found that the addition of an appropriate amount of Ca can refine the microstructure of the alloy; among them, the Mg-4.5Zn-4.5Sn-2Al-0.2Ca alloy has the best mechanical properties. Our research group has studied the Mg-Sn-Zn-Mn alloy, and found that the Mg-6Sn-4Zn-1Mn alloy has the best performance, and thus the Mg-6Sn-4Zn-1Mn-Al alloy and the Mg-6Sn-4Zn-1Mn-Ca alloy were chosen for study. However, the effect of the Al element on the Mg-6Sn-4Zn-1Mn-Ca alloy is still not fully understood. In order to study the effect of the Al element on the Mg-Zn-Sn-Mn-Ca alloy, and explore the potential properties of the Mg-Zn-Sn-Mn-Ca-xAl alloy, the different contents of Al are added to the Mg-6Zn-4Sn-1Mn-0.2Ca alloy, and the microstructure and properties of Mg-6Zn-4Sn-1Mn-0.2Ca-xAl alloy are investigated. It is expected that these studied alloys can be applied to the field of high-strength heat resistant deformation materials.

## 2. Experiment

The furnace temperature was set at 720 °C, and the mixed gas of CO_2_ and SF_6_ acted as the protective gas. Pure Mg (≥99.9 wt.%), Mg-4.10 wt.% Mn, pure Al (≥99.9 wt.%), pure Sn (≥99.9 wt.%), pure Zn (≥99.9 wt.%) and Mg-30 wt.% Ca were added successively. After the melting was completed, the ingot was poured into the mold to form. After that, the as-cast alloys were homogenized, that is, the as-cast alloy was placed in a heating furnace at 330 °C and keep at a constant temperature for 24 h. The homogenization treatment can eliminate the ingot’s ingot composition and microstructure inhomogeneity, and can preheat the ingot for plastic deformation. The plastic deformation process adopted in this paper was extrusion treatment, because compared with deformation processes such as forging and rolling, the extrusion treatment process is subjected to compressive stress in three directions at the same time, which can make the plasticity of the alloy play out to the maximum. In this experiment, the extrusion equipment was a 500 t horizontal extrusion press, the extrusion process was forward extrusion, and the diameter × length of the extrusion cylinder was φ 80 mm × 450 mm. The detailed extrusion parameters are shown in Table 1. After extrusion, solution treatment and aging treatment will further enhance the alloys’ mechanical properties. This solution treatment is adopted in this paper on 440 °C/2 h, and the aging treatment includes single-stage aging and two-stage aging, respectively. The detailed process is shown in Table 2.

In this paper, an U1tima IV X-ray diffractometer was chosen to identify the experimental alloys’ phases. The specific test parameters are as follows: the scanning angle was 10~90°, and the scanning speed was 4°/min. The microstructure of the alloy was observed by optical microscopy, scanning electron microscopy and transmission electron microscopy. The optical microscope used was an Olympus BX53M. The model of the scanning electron microscope was a JSM-6360, and the scanning electron microscope was combined with an Energy Dispersive Spectrometer (EDS) for point measurement, line scanning, and surface scanning at the same time. The EDS used was an Oxford INCA Energy 350. Secondary electron scanning electron microscope (SE-SEM) and back scattered electron scanning electron microscope (BSE-SEM) were also used. A FEI Tecnai G2 F20 Transmission Electron Microscope (TEM) was used, and the alloy sample was prepared according to the needs of the Bright field image (Bright field, BF) and the high-angle annular dark-field scanning transmission electron microscope (HAADF-STEM).

**Figure 1:**
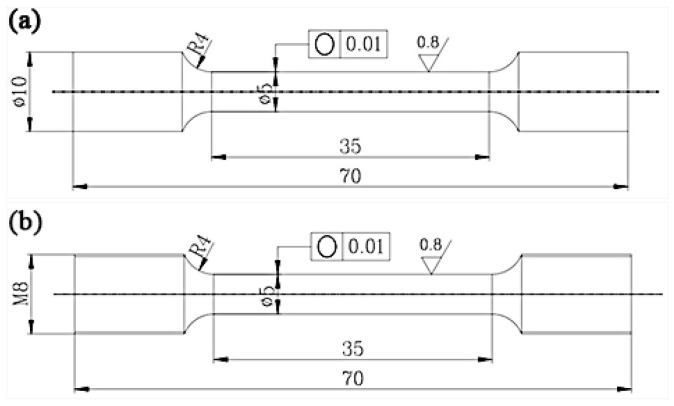
The schematic of the tensile sample at room and high temperature, (**a**) room temperature, (**b**) high temperature.

In order to evaluate the comprehensive properties of the alloy, the extrusion state and the aging state of the alloy are selected to carry out a unidirectional tensile test at room temperature. The room temperature performance test followed the ASTM B557M-02 standard. The experimental instrument used was a Xin think CMT-5105 microcomputer controlled electronic universal testing machine. The unidirectional tensile test was carried out at uniform speed, and the tensile rate was 3 mm/s. Before stretching, according to the GB228-2002 standard, the stretching sample was made into the stretching key in Figure 1a. On the basis of the room temperature tensile test of the developed alloy, the extruding alloy was further selected. According to the GB228-2002 standard, the tensile bond was made, as shown in Figure 1b. After that, the high temperature performance tests were carried out at 150 °C and 200 °C, respectively, using an Instron 3369 electronic universal material testing machine. The high temperature performance test followed the ASTM E21→92(1998) el standard. We adopted uniform unidirectional drawing, held the heat for 10min before drawing, and the drawing speed was 2 mm/min.

## 3. Results and Discussion

### 3.1. As-Cast Microstructure

One characteristic of the Mg alloy is its low density. In order to confirm whether the density of the studied alloy still meets the requirements of the light alloy, the density test was conducted on the as-cast ZTM641-0.2Ca-xAl (x = 0, 0.5, 1, 2) alloy, and the test results are shown in Table 3. It can be seen from the table that the density of the as-cast ZTM641-0.2Ca is 1.7666 g/cm^3^, the density of ZTM641-0.2Ca-0.5Al is 1.8240 g/cm^3^, and the density of ZTM641-0.2Ca-1Al is 1.8284 g/cm^3^. The density of ZTM641-0.2Ca-2Al is 1.8359 g/cm^3^. Generally speaking, the alloy density increases with the increase in Al content.

**Figure 2:**
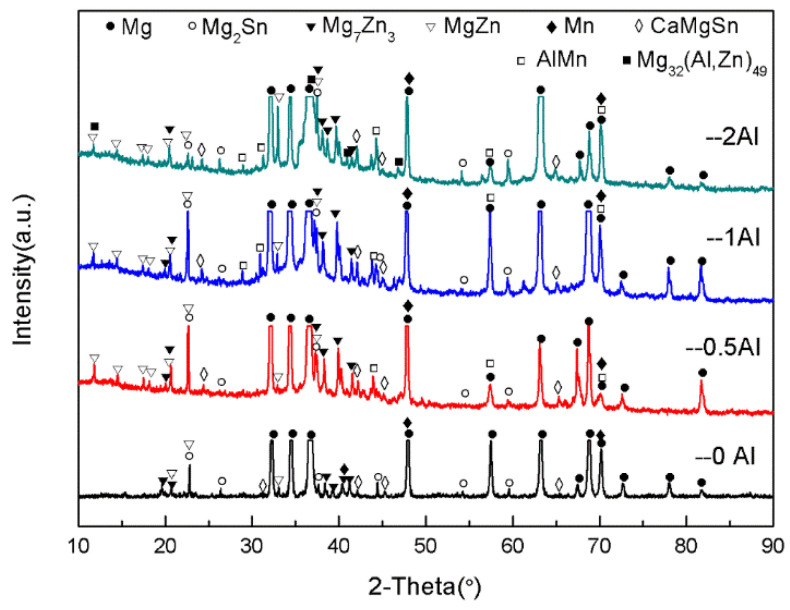
XRD patterns of as-cast ZTM641-0.2Ca-xAl (x = 0, 0.5, 1, 2) alloy.

Figure 2 is the XRD graph of the as-cast ZTM641-0.2Ca-xAl (x = 0, 0.5, 1, 2) alloy. From the figure, we can see that the ZTM641-0.2Ca alloy mainly has α-Mg, Mg_2_Sn, Mg_7_Zn_3_, MgZn, α-Mn and CaMgSn phases. As 0.5%Al is added to the alloy, the new diffraction peak of the AlMn phase appears. As the Al element continues to increase to 1%, the diffraction peak of the AlMn phase is significantly enhanced, which indicates that the number of AlMn phases in the ZTM641-0.2Ca-1Al alloy increases significantly. When the Al element continues to increase, a new diffraction peak of Mg_32_(Al,Zn)_49_ phases is found in the ZTM641-0.2Ca-2Al alloy, but the diffraction peak intensity of the AlMn phases is slightly weakened.

**Figure 3:**
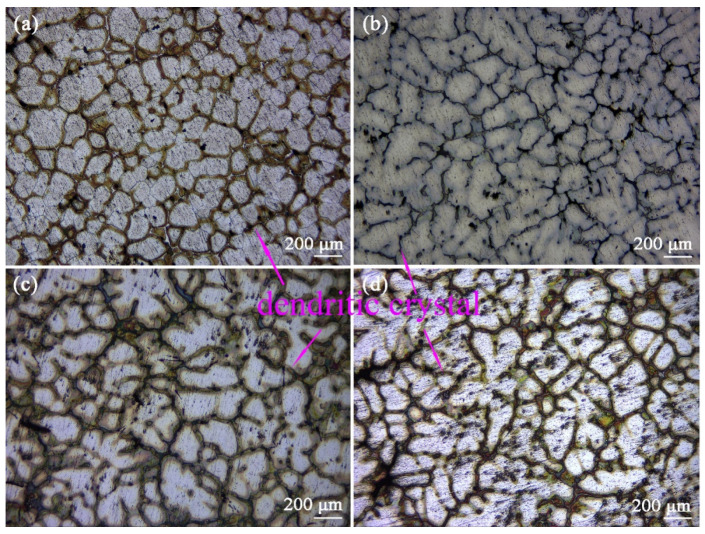
Optical micrographs of as-cast ZTM641-0.2Ca-xAl alloys: (**a**) x = 0; (**b**) x = 0.5; (**c**) x = 1; (**d**) x = 2.

Figure 3 is the Optical graphs of as-cast ZTM641-0.2Ca-xAl (x = 0, 0.5, 1, 2) alloys. From the figure, we can see that the as-cast microstructure mainly consists of an Mg matrix, dendrites, and a dispersed-distributed second phase. Compared with Figure 3a–d, the secondary growth of the dendrite is more sufficient, and the dendrite distribution is more dense with the addition of Al from 0 to 2%. This is due to the formation of the component supercooling of the Al element at the liquid-solid interface. When the amount of the Al element increases, the composition undercooling is obvious, which accelerates the growth rate of the dendrite tip, and then leads to the spacing decrease in the secondary dendrite.

**Figure 4:**
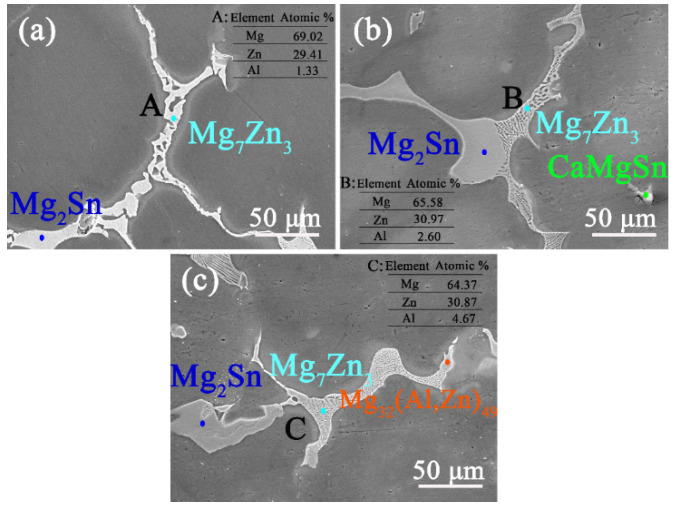
BSE-SEM micrographs of as-cast ZTM641-0.2Ca-xAl alloys: (**a**) x = 0.5; (**b**) x = 1; (**c**) x = 2.

Figure 4 shows the SEM graphs of the dendrites of the as-cast ZTM641-0.2Ca-xAl (x = 0.5, 1, 2) alloy. It can be seen that the dendrite of the ZTM641-0.2Ca-0.5Al alloy mainly has two phases, one being the Mg_2_Sn phase and the other the Mg_7_Zn_3_ phase, in which the Mg_7_Zn_3_ phase shows a network structure. As the Al element increases to 1%, the type for the dendrite phases does not change, but the morphology of the Mg_7_Zn_3_ phase changes significantly, and its network structure is obviously refined, as depicted in Figure 4b. When the Al content continues to increase, the morphology and type of dendrite phase are changed for the ZTM641-0.2Ca-2Al alloy. It mainly has an Mg_2_Sn phase, an Mg_7_Zn_3_ phase, and an Mg_32_(Al,Zn)_49_ phase. Meanwhile, the network structure of the Mg_7_Zn_3_ phase is further refined, as shown in Figure 4c. The gradual refinement of the network structure of the Mg_7_Zn_3_ phase may result from the aliquation of the Al element on the Mg-Zn eutectic compound. According to the EDS data for point A, B and C, The Mg_7_Zn_3_ phase contains different Al content, and the greater the Al element, the finer the network structure.

### 3.2. As-Homogenized Microstructure

**Figure 5:**
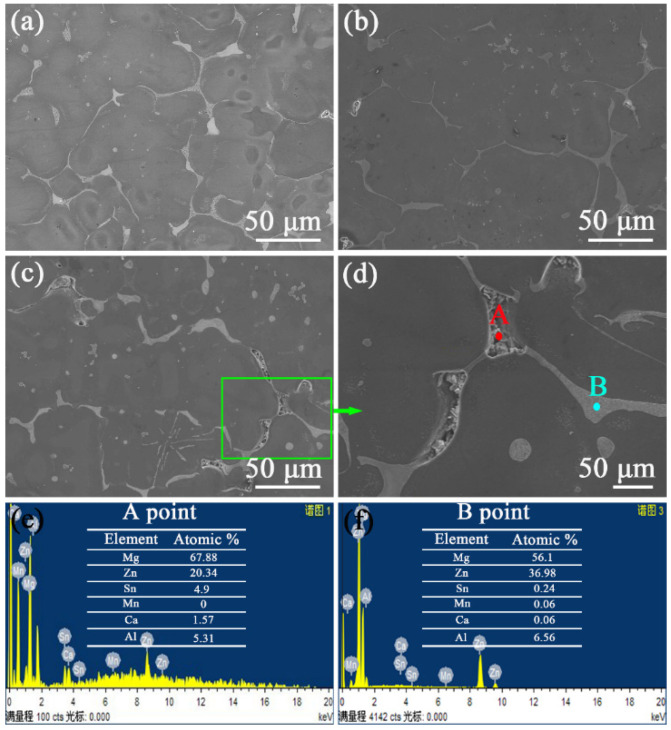
BSE-SEM micrographs of as-homogenized ZTM641-0.2Ca-xAl alloys: (**a**) x = 0.5, (**b**) x = 1, (**c**) x = 2, (**d**) Enlargement of circular rectangular part in (**c**); (**e**,**f**) Corresponding EDS results of points in (**d**).

Figure 5 shows the SEM graphs for the as-homogenized ZTM641-0.2Ca-xAl (x = 0.5, 1, 2) alloys. It shows that the microstructure is made up of the Mg matrix, the dendritic phase, and the dispersed second phase. Compared to an as-cast microstructure, the as-homogenized dendritic phases are of discontinuous distribution. This indicates that a part of the dendrites has been dissolved in the matrix. Figure 5d is an enlargement of the green box in Figure 5c. As we can see in this graph that a large number of white fine points are distributed around the dendrite, showing a gray transition shape, this indicates that a part of the dendrite structure is melted by the heat treatment, while the white fine points are residual eutectic compounds.

In order to determine the composition of the dendrite structure, an EDS analysis was conducted, and the results are shown in Figure 5e,f. We can see that the composition of point A is mainly Mg, Zn, Sn, Ca and Al, while point B mainly contains Mg and Zn elements, mixed with a small amount of the Al element. Combined with the XRD results, it can be determined that point A and B are of the Mg_7_Zn_3_ phase.

### 3.3. As-Extruded Microstructure

Table 4 shows the density of the as-extruded ZTM641-0.2Ca-xAl (x = 0, 0.5, 1, 2) alloy. It can be seen from the table that the density of as-extruded ZTM641-0.2Ca-0.5Al is 1.8318 g/cm^3^. The density of as-extruded ZTM641-0.2Ca-1Al and ZTM641-0.2Ca-2Al is 1.8469 g/cm^3^ and 1.8641 g/cm^3^, respectively. The density increases with the increase of the Al content, which is the same as that of the as-cast alloy. However, the density of the as-extruded alloy is slightly higher. This is because after the homogenization and extrusion treatment, the as-extruded composition is more uniform and the microstructure is more closely distributed, which can be confirmed in the subsequent microstructure analysis.

**Figure 6:**
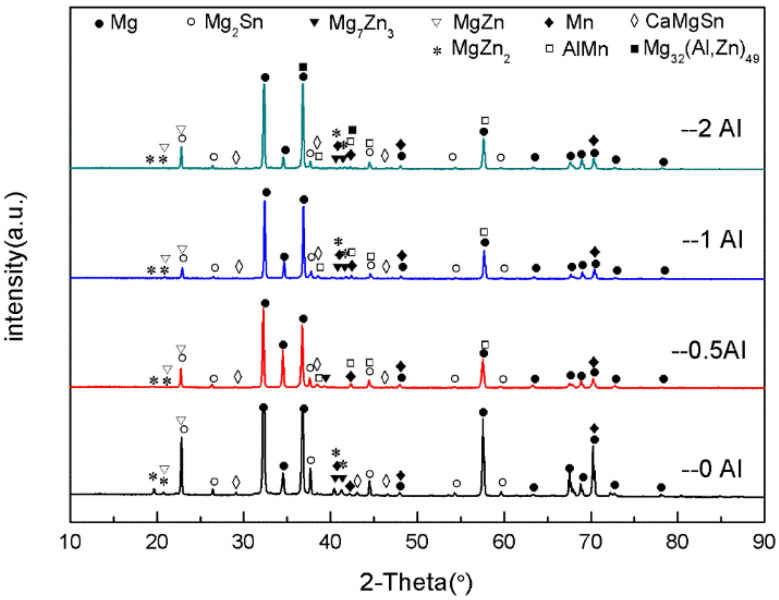
XRD patterns of as-extruded ZTM641-0.2Ca-xAl (x = 0, 0.5, 1, 2) alloy.

Figure 6 shows the XRD patterns for the as-extruded ZTM641-0.2Ca-xAl (x = 0, 0.5, 1, 2) alloy. According to the results, the as-extruded ZTM641-0.2Ca-xAl alloys mainly consist of the α-Mg, Mg_2_Sn, Mg_7_Zn_3_, MgZn, α-Mn, CaMgSn, MgZn_2_, AlMn, and Mg_32_(Al,Zn)_49_ phases. Unlike the as-cast alloys, these fine diffraction impurity peaks in the as-extruded alloys are significantly reduced after homogenization treatment and extrusion plastic deformation. At the same time, a new diffraction peak of the MgZn_2_ phase appears in the as-extruded alloy, and the diffraction peak intensity of the MgZn_2_ phases is significantly weakened after the addition of the Al element, which may be due to the combination of Al with the Mg and Zn elements to generate the Mg_32_(Al,Zn)_49_ phase, which consumes part of Zn element. The variation of diffraction peak intensity of the other phases is similar to that of an as-cast alloy.

**Figure 7:**
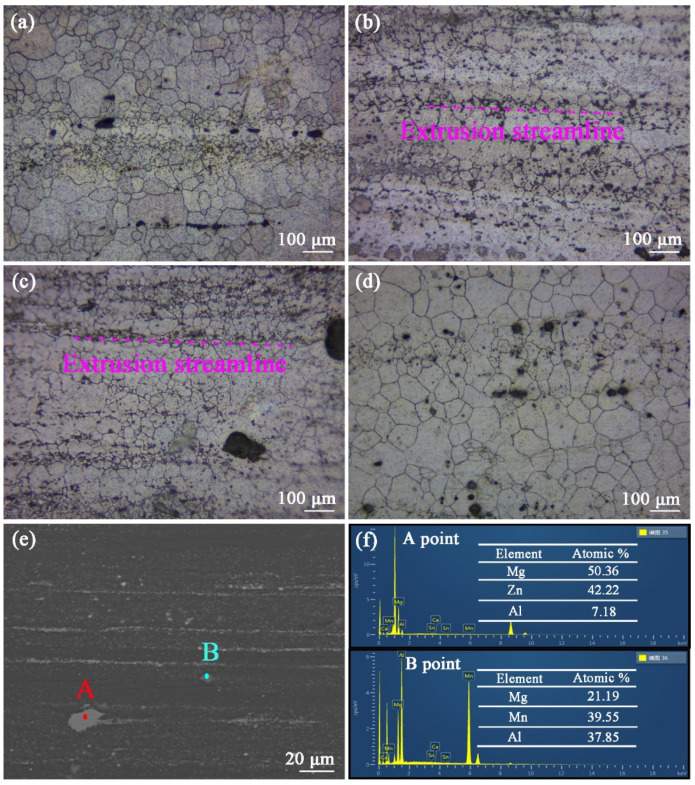
Optical micrographs of as-extruded ZTM641-0.2Ca-xAl alloys: (**a**) x = 0; (**b**) x = 0.5; (**c**) x = 1; (**d**) x = 2; (**e**) BSE-SEM micrographs and (**f**) EDS results of as-extruded ZTM641-0.2Ca-2Al alloys.

Figure 7a–d are the longitudinal metallographic for the as-extruded ZTM641-0.2Ca-xAl (x = 0, 0.5, 1, 2) alloys. It shows that dynamic recrystallization occurs after the hot extrusion treatment, and these bulk second phases are broken into small particles; these small particles are streamlined distribution. Compared with figure (a), (b), (c) and (d), it was determined that the recrystallized grain size of the ZTM641-0.2Ca-1Al alloy was the smallest. According to the research of Humphreys F J [19], the large second phase will cause lattice distortion, produce large-angle grain boundaries, promote the crystal grains nucleation, increase the number of crystal grains, and significantly reduce the crystal grain diameter. For studying these second phases of the as-extruded alloys, the as-extruded ZTM641-0.2Ca-2Al alloy was chosen for SEM observation. The observation results are shown in Figure 7e. It can be seen that the white second phase particles are distributed into the matrix with a streamlined distribution, and some large second phases are also observed. Through the EDS results in Figure 7f, as we can see that point A mainly contains three elements: Mg, Zn and Al. According to the XRD pattern in Figure 6, the second phase can be determined as the Mg_32_(Al,Zn)_49_ phase. Point B mainly contains the three elements of Mg, Al and Mn, and Al:Mn = 1:1. Combined with the XRD pattern, the second phase can be determined as the AlMn phase.

**Figure 8:**
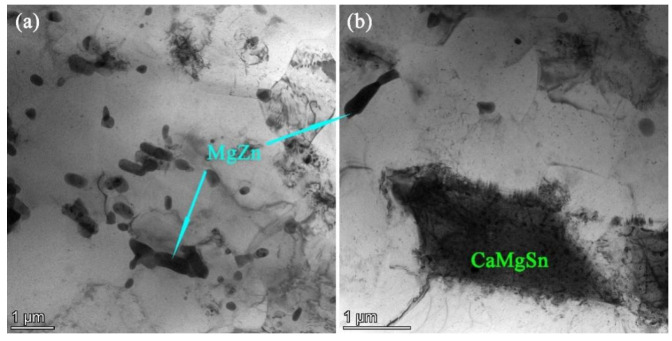
TEM micrographs of as-extruded ZTM641-0.2Ca-2Al alloy.

Figure 8 shows the TEM micrographs of the as-extruded ZTM641-0.2Ca-2Al alloy. As shown in Figure 8a, the microstructure of the as-extruded ZTM641-0.2Ca-2Al alloy contains some rod-like phases of about 1 μm in diameter, and the distribution is relatively concentrated. The aggregation of rod-like phases may be due to the large number of boundary surfaces of the rod-like phases, and the aggregation growth can reduce part of the interface. According to the minimum energy criterion, the decrease of interface energy can make the system tend to a more stable state. The EDS results and XRD patterns confirm that these rod-like phases are MgZn phases. The rod-like MgZn phase is also observed in Figure 8b. Through the EDS results, the CaMgSn phase is observed in the dislocation pile-up. This indicates that the Ca element and Sn element are enriched here. This is due to the lower energy required for the movement of atoms in dislocations, which makes it easier for Ca to combine with the Sn and Mg atoms. Therefore, the CaMgSn phase is nucleated and precipitated with dislocation and dislocation entanglement.

### 3.4. Solution-Treated and Aged Microstructures

**Figure 9:**
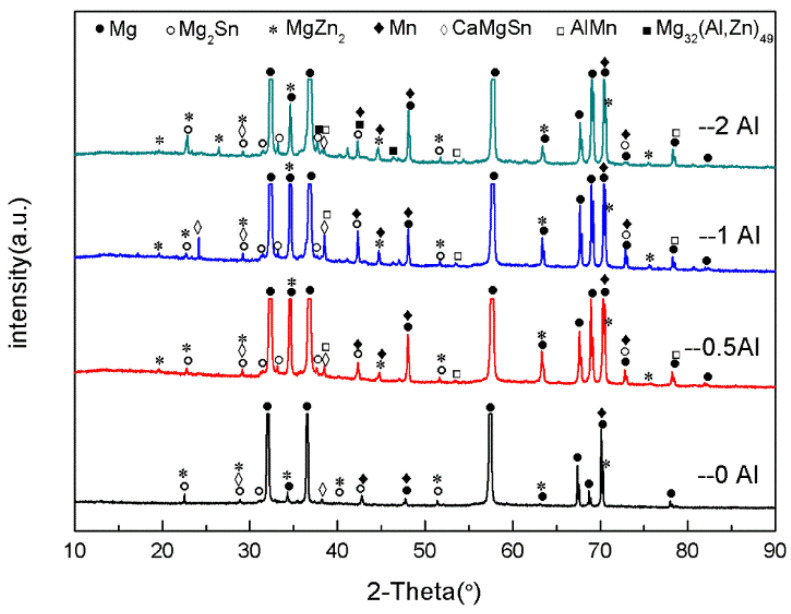
XRD patterns of solid solution ZTM641-0.2Ca-xAl (x = 0, 0.5, 1, 2) alloys.

The phases of the solid solution ZTM641-0.2Ca-xAl (x = 0, 0.5, 1, 2) alloy are analyzed by XRD patterns. The results are shown in Figure 9. Among them, the solid solution ZTM641-0.2Ca alloy mainly consists of α-Mg, Mg_2_Sn, MgZn_2_, α-Mn and CaMgSn phases. As 0.5%Al is added to the alloy, the ZTM641-0.2Ca-0.5Al alloy is α-Mg, Mg_2_Sn, MgZn_2_, α-Mn, CaMgSn and AlMn phases, and the Al element is mainly used to form the AlMn phase. As the Al content increases to 1%, the phase type does not change. As the Al element continues to increase to 2%, the phase of ZTM641-0.2Ca-2Al changes into the α-Mg, Mg_2_Sn, MgZn_2_, α-Mn, CaMgSn, AlMn and Mg_32_(Al,Zn)_49_ phases. Due to the further increase of the Al content, the Al element is mainly used to form the AlMn phase and the Mg_32_(Al,Zn)_49_ ternary phase. Compared with the as-extruded alloys, the diffraction peaks of the Mg_7_Zn_3_ and MgZn phases are not detected. This indicates that these two phases all melt into the matrix, and the Zn element is mainly combined with Mg and Al to form the MgZn_2_ phase and the Mg_32_(Al,Zn)_49_ phase. Comparing the several curves, it is found that when the Al element increases, the diffraction peak intensity of the AlMn phase increases significantly.

**Figure 10:**
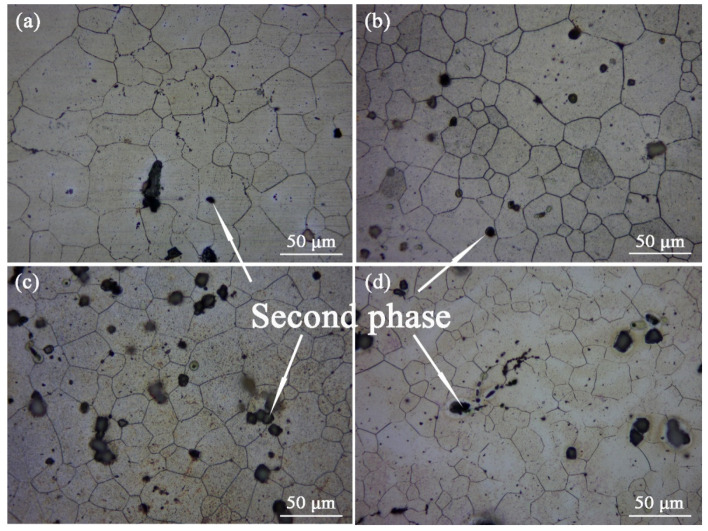
Optical micrographs of solid solution ZTM641-0.2Ca-xAl alloys: (**a**) x = 0; (**b**) x = 0.5; (**c**) x = 1; (**d**) x = 2.

Figure 10 shows the optical micrographs of the solid solution ZTM641-0.2Ca-xAl (x = 0, 0.5, 1, 2) alloy. According to the figure, the microstructure for the solid solution alloy includes the Mg matrix, grain, and second phases. After this solution treatment, the recrystallized grain grows further, and the average grain size decline with the Al element rises, although the change is not very obvious, which indicates that the Al element plays a certain role in grain refinement for the alloys. The grain refinement may be due to the combination of the Al element and the Mn element to form the AlMn phase. The AlMn phase preferably precipitates and concentrates at the front-end α-Mg phase, which pins the grain boundary and hinders the grain growth. For the ZTM641-0.2Ca alloy, the majority of second phases have been dissolved into the matrix, as shown in Figure 10a. When 0.5%Al is being added, some black second phase with a diameter of about 5 μm is observed in the microstructure, a large number of them are distributed on the grain boundary, and a few are distributed in the grain. As the Al element increases to 1%, the number of second phase particles rise, and the diameters of these particles rise to about 10 μm. All of them are distributed on the grain boundaries, and some of them are of an aggregated distribution. When the Al content continues to increase to 2%, the second phase particle continues to grow.

**Figure 11:**
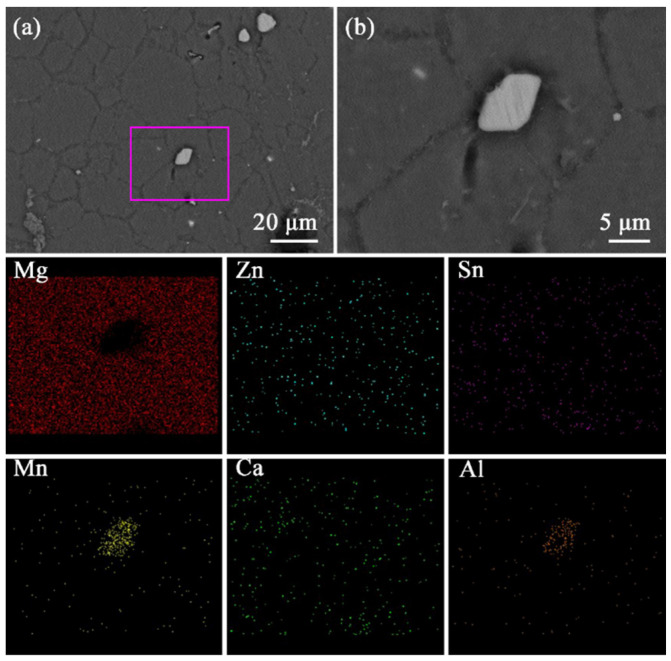
BSE-SEM micrographs and mapping of solid solution ZTM641-0.2Ca-0.5Al alloys: (**a**) BSE-SEM micrograph, (**b**) Enlargement of the rectangular part in (**a**).

To observe the microstructure and morphology for solid solution alloys from a clearer perspective, the solid solution ZTM641-0.2Ca-0.5Al alloy is selected for scanning electron microscope observation. The observation results are shown in Figure 11a. We can see that the solid solution structure has substrate and second phase particles, and the particle sizes are not unique, among which the particles with a small diameter are point-like. They are mainly located in the grain boundary, although a small portion of them are located in the grain interior. The second phase, which is larger in size, is angular and massive, mainly distributed on the grain boundary. Figure 10b is the red box’s magnification in Figure 10a. We can clearly observe that these second phases of the bright white angular block are located at the intersection of several grain boundaries. The grain diameter is about 5 μm. In order to determine the specific composition of the second phase particles in the figure, the region shown in Figure 10b was scanned by the energy spectrum, as shown in Figure 11. It can be observed that Mg mainly exists in the matrix. Zn appears in the matrix and second phases. Sn elements are also segregated in the matrix and second phases. The Mn element appears to be segregated in the second phase and disperses in the matrix. The Ca elements are uniformly distributed in the matrix and second phases. This distribution of Al elements in the matrix is uniform, and there is segregation in the second phase particles. In general, these second phases of the angular block are AlMn phases.

**Figure 12:**
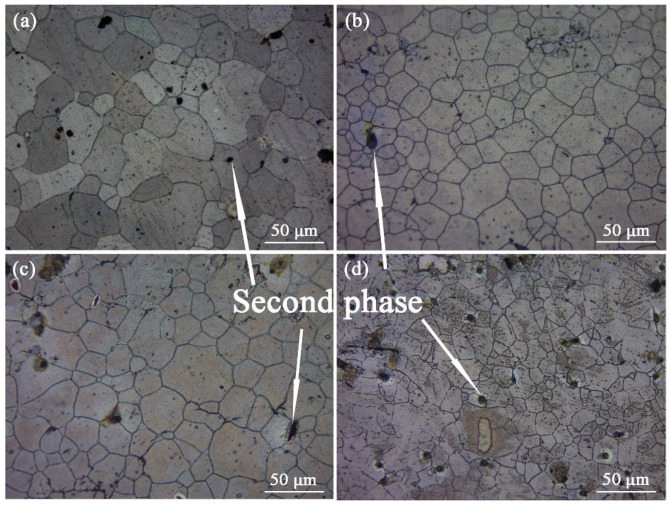
Optical micrographs of two-stage aged ZTM641-0.2Ca-xAl alloys: (**a**) x = 0; (**b**) x = 0.5; (**c**) x = 1; (**d**) x = 2.

After solid solution treatment, the ZTM641-0.2Ca-xAl (x = 0, 0.5, 1, 2) alloy is subjected to artificial aging treatment. Because there is no obvious difference in the microstructure between one-stage and two-stage aged alloys, we selected the two-stage aged alloy as that representing the microstructure analysis in this paper. Figure 12 is the optical micrograph of the two-stage aged ZTM641-0.2Ca-xAl (x = 0, 0.5, 1, 2) alloy. We can see that these second phase particles at the microstructure are less for the ZTM641-0.2Ca-0.5Al alloy. The second phase particles increased significantly for the ZTM641-0.2Ca-1Al alloy, most of which are located on the grain boundary, as shown in Figure 12c. When the Al element increases to 2%, the more second phases particles that appear in the microstructure, the more diffuse is the distribution, the majority of them are located in grain boundaries, and a few of them are distributed in grains. Compared with Figure 12a–d, it was found that the grain size declines when the Al element increases, and the grain size of the ZTM641-0.2Ca-2Al alloy is the smallest.

**Figure 13:**
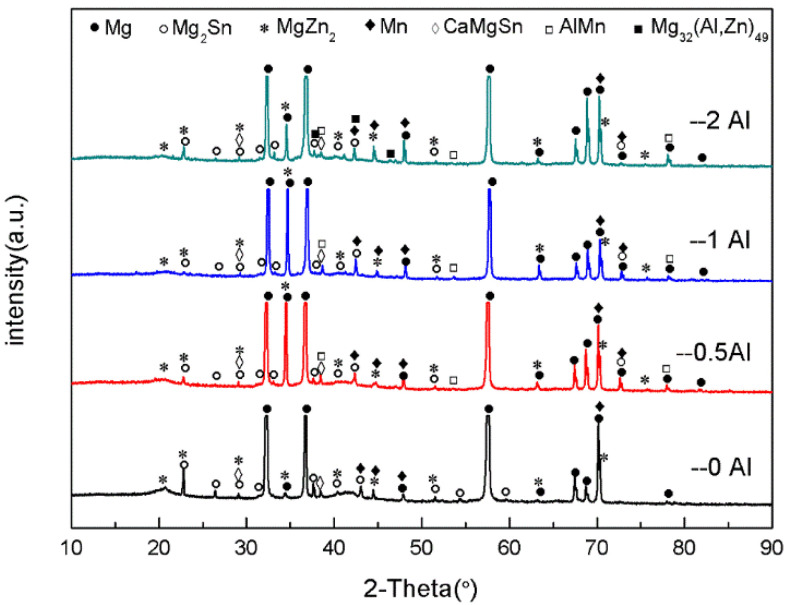
XRD patterns of two-stage aged ZTM641-0.2Ca-xAl (x = 0, 0.5, 1, 2) alloy.

Figure 13 shows the XRD patterns of the two-stage aged ZTM641-0.2Ca-xAl (x = 0, 0.5, 1, 2) alloy. From this figure, we can see that the phase composition of the two-stage aged alloy are mainly the α-Mg, Mg_2_Sn, MgZn_2_, α-Mn, CaMgSn, AlMn and Mg_32_(Al,Zn)_49_ phases. The addition of Al mainly forms the AlMn phase and the Mg_32_(Al,Zn)_49_ phase with the other elements in the alloy. The variation of other precipitated phases is similar to that of the solid solution alloy, but these diffraction peaks of Mg_2_Sn and MgZn_2_ phases for the two-stage aged alloy are obviously wide, which indicates that the particle size for these two precipitated phases is small.

**Figure 14:**
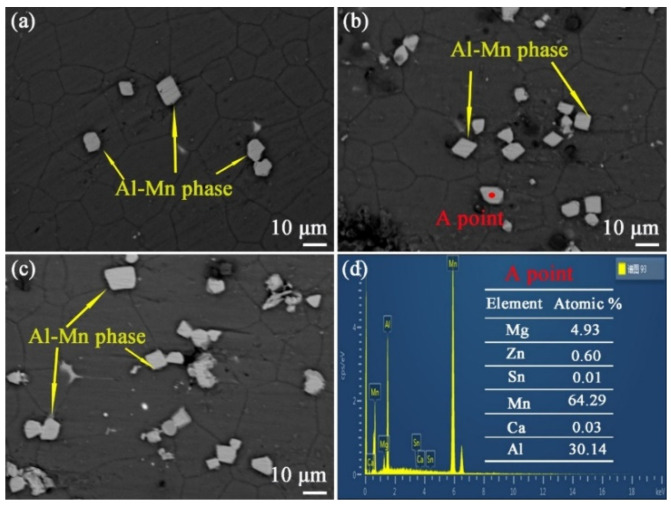
BSE-SEM micrographs of two-stage aged ZTM641-0.2Ca-xAl alloys: (**a**) x = 0.5, (**b**) x = 1, (**c**) x = 2; (**d**) The corresponding EDS result of the A point.

To clearly observe the microstructure of the aged alloy, the two-stage aged ZTM641-0.2Ca-xAl (x = 0.5, 1, 2) alloy is observed in these SEM images. As shown in Figure 14, the microstructure of the alloys mainly consists of the Mg matrix and second phases. Among them, the ZTM641-0.2Ca-0.5Al alloy has a large second phase with a diameter of about 10 μm and a blocky morphology. In addition, most of these second phases are located on grain boundaries, while a few are located at the grain. For the ZTM641-0.2Ca-1Al alloy, the number of large second phase particles of these alloys increases, while the particles’ morphology and distribution are similar to the ZTM641-0.2Ca-0.5Al alloy. For the ZTM641-0.2Ca-2Al alloy, the number of second phase particles of these alloys increases further, and the morphology is still angular and blocky. It can be seen in Figure (c) that these second phase particles are mainly located on the grain boundary.

In order to determine the composition of these second phases, EDS testing was chosen, and these results are shown at Figure 14d. As shown in the graph, the elemental composition of the second phase in point A is mainly Mg, Al and Mn. Due to the second phase being located on the matrix, the Mg element will inevitably exist in the EDS results. Therefore, this second phase is the Al-Mn phase. Compared with Figure 10 and Figure 12, the aged alloy has more second phase particles. This is due to most of the Al and Mn atoms being dissolved into the matrix during the solid solution process. When the alloy is subjected to an aging treatment, the Al and Mn atoms will continue to precipitate with the form of Al-Mn phase. It is worthy of note that in the EDS result in Figure 14d, the atomic ratio of the precipitated phase is Al:Mn = 1:2, but the XRD graph in Figure 13 shows that the Al-Mn phase is the AlMn phase. These results conflict with each other. According to the previous literature [20], some researchers think that the Al-Mn phase is the Al_8_Mn_5_ phase. In this paper, this phase was tentatively named the Al-Mn phase after thorough consideration.

### 3.5. Mechanical Properties

**Figure 15:**
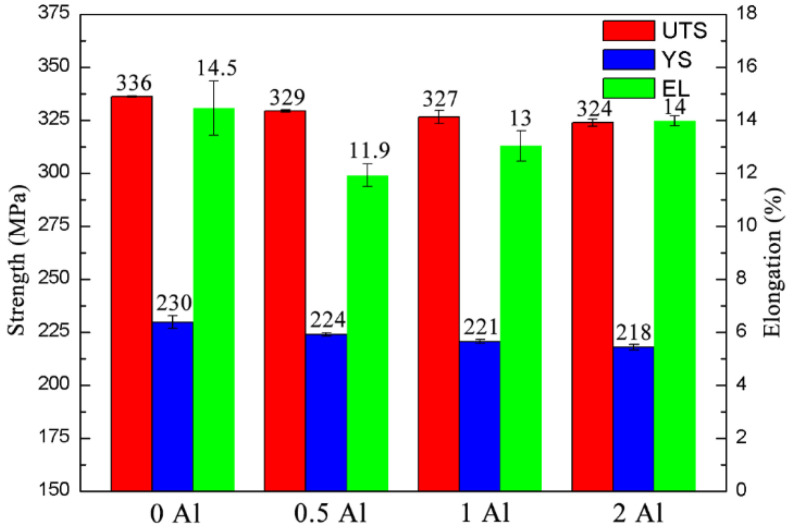
Mechanical properties of as-extruded ZTM641-0.2Ca-xAl alloys tested at room temperature.

Figure 15 shows the mechanical properties of the as-extruded ZTM641-0.2Ca-xAl (x = 0, 0.5, 1, 2) alloy at room temperature. As can be seen, the as-extruded ZTM641-0.2Ca alloy has the highest comprehensive mechanical property; that is, the UTS, YS and elongation are 336 MPa, 230 MPa and 14.5%. According to our previous research, the UTS, YS and the elongation of the Mg-6Sn-4Zn-1Mn alloy are 328 MPa, 255 MPa and 10.76%, respectively. When 0.5% Al was added, the properties of the alloy decreased slightly, with the UTS, YS and elongation at 329 MPa, 224 MPa and 11.9%, respectively. When the Al content increases to 1%, the UTS and YS decrease to 327 MPa and 221 MPa, but the elongation increases to 13%. When the Al content is 2%, the UTS is 324 MPa, the YS is 218 MPa, and the elongation is 14%. In general, the properties of the as-extruded ZTM641-0.2Ca-xAl (x = 0, 0.5, 1, 2) alloy decreased slightly with the increase of Al content, but the overall difference was not significant. The reason for this variation trend is that the Al element was added to the alloy and combined with the Mn element in the alloy to form the AlMn phase. According to the previous microstructure analysis, the higher Al content, the larger the second phase, and the worse binding force with the matrix. When the alloy is deformed, the coordinating deformation ability of the alloy is poor, resulting in a decrease in the strength of the alloy.

**Figure 16:**
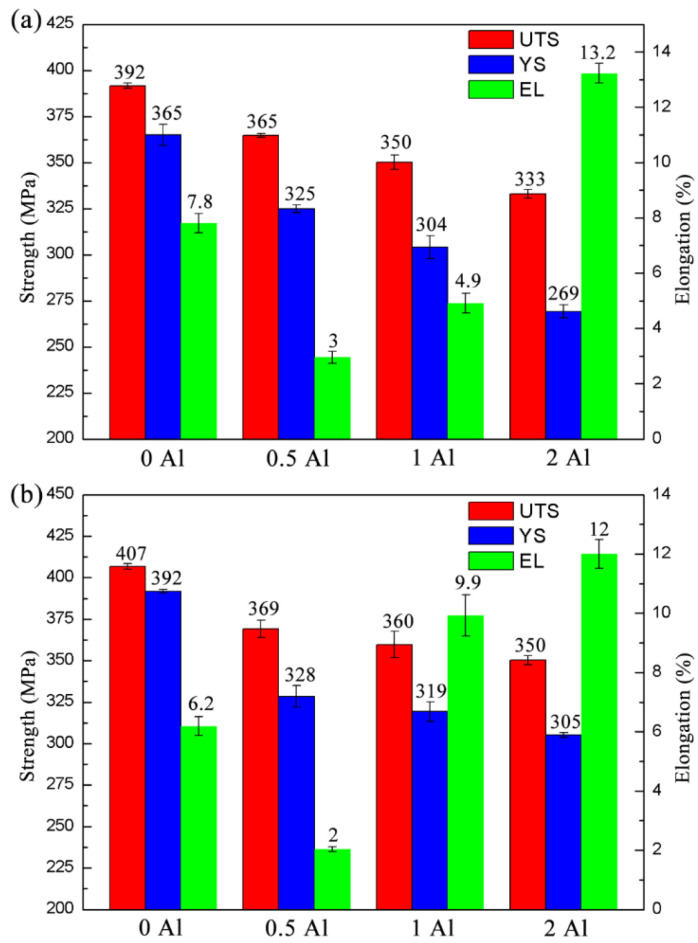
Mechanical properties of aged ZTM641-0.2Ca-xAl alloys tested at room temperature: (**a**) one-stage aging; (**b**) two-stage aging.

Figure 16 shows the strength and plasticity of the aged ZTM641-0.2Ca-xAl (x = 0, 0.5, 1, 2) alloy at room temperature, and Figure (a) and Figure (b) correspond to the one-stage aging and two-stage aging, respectively. From Figure (a), we can see that the one-stage aged ZTM641-0.2Ca alloy has the highest strength, with the UTS and YS being 392 MPa and 365 MPa, respectively. According to our previous research [21], the UTS and YS of the one-stage aged ZTM641-0.2Al alloy are 382 MPa and 352 MPa, and the UTS and YS of one-stage aged ZTM641-0.5Al alloy are 350 MPa and 299 MPa. When 0.5% Al is added to the alloy, the strength and plasticity show a decreasing trend, while the UTS and YS are 365 MPa and 325 MPa, respectively. When the Al content increases to 1%, the UTS and YS continually decrease to 350 MPa and 304 MPa, respectively. In addition, the UTS and YS of the ZTM641-0.2Ca-2Al alloy are further reduced to 333 MPa and 269 MPa, respectively. On the other hand, with the gradual increase of the Al content, the elongation of the alloy gradually increases. When the Al content increases to 2%, the elongation increases significantly, to 13.2%. For the two-stage aged alloy, the variation of properties is consistent with that of the one-stage aged alloy. As shown in Figure 16b, the two-stage aged ZTM641-0.2Ca alloy also has the highest strength, with UTS and YS at 407 MPa and 392 MPa, respectively. According to our previous research, the UTS and YS of the two-stage aged ZTM641-0.2Al alloy are 384 MPa and 360 MPa. When 0.5%Al is added to the alloy, the alloy strength decreases slightly, and the UTS and YS are 369 MPa and 328 MPa, respectively. When the Al content continues to increase, the strength of the alloy also continues to decrease. When the Al content is 2%, the UTS and YS of the alloy decreases to 350 MPa and 305 MPa. When the Al atom content is 2%, the elongation of the alloy is the best, at 12%. The gradual improvement of the plasticity may be due to the more Al atoms that are dissolved into the matrix with the increase of Al content, which effectively reduces the stacking fault energy of the alloy. Due to the low stacking fault energy of the alloy, dislocation cells do not form easily, which effectively prevents the nucleation and growth of cracks, and improves the plasticity of the alloy [22]. As for the alloy strength gradually decreasing, this may be due to the large size of the Al-Mn phase in the alloy. On the one hand, the large size of the second phase reduces the coordinated deformation ability of the matrix. On the other hand, it can be clearly observed from Figure 14 that the large size of the Al-Mn phase is mainly distributed on the grain boundary. With the increase of Al content, the amount of the Al-Mn phase on the grain boundary also increased. Due to the size of the second phase particles on the grain boundary affecting the plastic deformation ability, when the size and number of the second phase particles on the grain boundary are larger, the cracks are more likely to appear in the alloy, and the corresponding strength is lower. Therefore, the strength of the ZTM641-0.2Ca-2Al alloy is the lowest.

**Figure 17:**
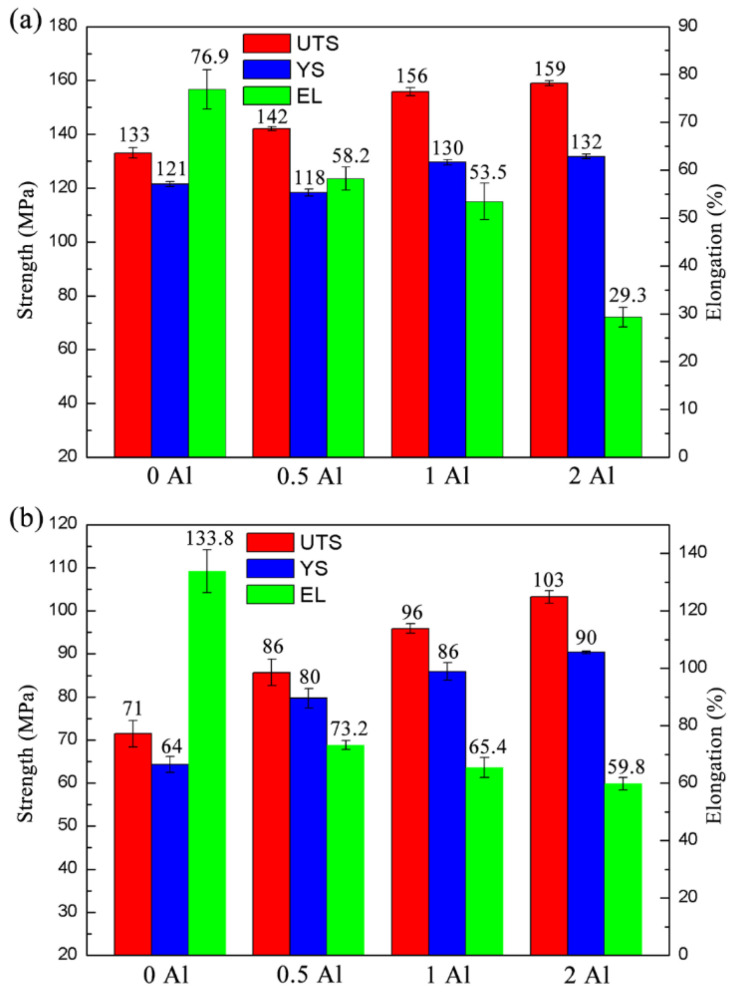
Mechanical properties of as-extruded ZTM641-0.2Ca-xAl alloys tested at high temperature: (**a**) 150 °C; (**b**) 200 °C.

Figure 17a,b show the strength and plasticity of the as-extruded ZTM641-0.2Ca-xAl (x = 0, 0.5, 1, 2) alloy at 150 °C and 200 °C, respectively. It can be seen from Figure (a) that the UTS and YS of the ZTM641-0.2Ca-xAl alloy gradually increases with the addition of the Al element. Among them, the strength of the ZTM641-0.2Ca-2Al alloy i the highest, and the UTS and YS are 159 MPa and 132 MPa, respectively. However, the elongation of the ZTM641-0.2Ca-xAl alloy decreases gradually with the addition of Al; the elongation of ZTM641-0.2Ca-2Al alloy is only 5%. According to Figure (b), the variation trend of the mechanical properties at 200 °C is consistent with that at 150 °C. The UTS and YS of the ZTM641-0.2Ca-2Al alloy are the highest, at 103 MPa and 90 MPa. However, the elongation is lower, at 59.8%.

According to the previous as-extruded microstructure, it can be seen that the Al element is added to the alloy and combines with the Mn element to generate the AlMn phase. The AlMn phase is a high-melting phase with a melting point of 670 °C. When the alloy is deformed under high temperature, the AlMn phase can pin the grain boundaries, hindering dislocation slip. According to metal theory [23], when the alloy is subjected to stress and deformation at high temperatures, if the size of second phase is large, the dislocation cannot cut through the second phase, but can only bypass the second phase. In this process, the dislocation must overcome the resistance caused by the bending dislocation tension. Specifically, the shear stress required by the dislocation bypass phase is shown in the following formula:

$$\tau =G\frac{b}{\lambda }$$

where G is the tangent modulus of elasticity, *b* is the Berkovian vector of dislocations, and λ is the distance between the dislocation and the precipitate. Based on the previous microstructure analysis, when the Al content increases from 0.5% to 1% and 2%, the number and size of the second phase also increases correspondingly, and the required shear stress also increases, which improves the strength. However, due to the AlMn phase being a brittle phase, the higher content of the AlMn phase will inevitably lead to the deterioration of the plasticity.

## 4. Conclusions

In this paper, the Al element was added to the ZTM641-0.2Ca alloy, and the microstructure and mechanical properties of the ZTM641-0.2Ca-xAl (x = 0, 0.5, 1, 2) alloy were explored by means of XRD, OM, SEM, TEM, EDS and a unidirectional tensile experiment. The conclusions obtained are summarized as follows:

(1) The ZTM641-0.2Ca-xAl (x = 0.5, 1, 2) alloy is mainly composed of the α-Mg, Mg_2_Sn, Mg_7_Zn_3_, MgZn, MgZn_2_, α-Mn, CaMgSn, AlMn and Mg_32_(Al,Zn)_49_ phases. When the Al element is added to the alloy, the AlMn and Mg_32_(Al,Zn)_49_ phases are formed.
(2) When the Al element is added to the ZTM641-0.2Ca alloy, the alloy grains are refined, and the angular bulk AlMn phase is formed, which is mainly distributed at the grain boundaries. With the increase in Al element content, the amount of the AlMn phase increases, but the morphology does not change.
(3) At room temperature, the higher content of Al can improve the elongation of the aged alloy, and the two-stage aged ZTM641-0.2Ca-2Al alloy has the highest elongation, at13.2%. At high temperatures, the Al element can improve the strength, and the strength of the as-extruded ZTM641-0.2Ca-2Al alloy at 150 °C is the highest, with a UTS and YS of 159 MPa and 132 MPa, respectively.

## Figures and Tables

**Table 1:** Extrusion parameters for the studied alloys.

Test Materials	Billet Temperature (°C)	Extrusion Chamber Temperature (°C)	Mold Hole Diameter (mm)	Extrusion Speed (m/min)	Extrusion Ratio
Mg-6Zn-4Sn-1Mn-0.2Ca-xAl	360	350	16	2	25

**Table 2:** Heat treatment parameters for the studied alloys.

Type	Solution Treatment	Aging Treatment
T4		—
T4+single aging	440 °C/2 h	180 °C/12 h
T4+double aging		90 °C/24 h + 180 °C/8 h

**Table 3:** The density of as-cast ZTM641-0.2Ca-xAl alloys.

Alloys	ZTM641-0.2Ca	ZTM641-0.2Ca-0.5Al	ZTM641-0.2Ca-1Al	ZTM641-0.2Ca-2Al
Density (g/cm^3^)	1.7666	1.8240	1.8284	1.8359

**Table 4:** The density of as-extruded ZTM641-0.2Ca-xAl alloys.

Alloys	ZTM641-0.2Ca	ZTM641-0.2Ca-0.5Al	ZTM641-0.2Ca-1Al	ZTM641-0.2Ca-2Al
Density (g/cm^3^)	1.7889	1.8318	1.8469	1.8641

## Data Availability

Data will be made available on request.
